# Topical Probiotics: More Than a Skin Deep

**DOI:** 10.3390/pharmaceutics14030557

**Published:** 2022-03-03

**Authors:** Mohammed Habeebuddin, Ranjith Kumar Karnati, Predeepkumar Narayanappa Shiroorkar, Sreeharsha Nagaraja, Syed Mohammed Basheeruddin Asdaq, Md. Khalid Anwer, Santosh Fattepur

**Affiliations:** 1 Department of Biomedical Sciences, College of Medicine, King Faisal University, Al-Hofuf 31982, Saudi Arabia; hmohammed@kfu.edu.sa (M.H.); pshiroorkar@kfu.edu.sa (P.N.S.); 2 Department of Chemistry, College of Science, King Faisal University, Al-Hofuf 31982, Saudi Arabia; rkarnati@kfu.edu.sa; 3 Department of Pharmaceutical Sciences, College of Clinical Pharmacy, King Faisal University, Al-Hofuf 31982, Saudi Arabia; 4 Department of Pharmaceutics, Vidya Siri College of Pharmacy, Off Sarjapura Road, Bangalore 560035, India; 5 Department of Pharmacy Practice, College of Pharmacy, AlMaarefa University, Dariyah, Riyadh 13713, Saudi Arabia; sasdaq@gmail.com; 6 Department of Pharmaceutics, College of Pharmacy, Prince Sattam Bin Abdulaziz University, Al-Alkharj 11942, Saudi Arabia; mkanwer2002@yahoo.co.in; 7 School of Pharmacy, Management and Science University, Seksyen 13, Shah Alam 40100, Malaysia

**Keywords:** probiotics, inflammatory, microorganisms, dermatitis

## Abstract

Skin, an exterior interface of the human body is home to commensal microbiota and also acts a physical barrier that protects from invasion of foreign pathogenic microorganisms. In recent years, interest has significantly expanded beyond the gut microbiome to include the skin microbiome and its influence in managing several skin disorders. Probiotics play a major role in maintaining human health and disease prevention. Topical probiotics have demonstrated beneficial effects for the treatment of certain inflammatory skin diseases such as acne, rosacea, psoriasis etc., and also found to have a promising role in wound healing. In this review, we discuss recent insights into applications of topical probiotics and their influence on health and diseases of the skin. Patents, commercially available topical probiotics, and novel probiotic impregnated fabrics have been emphasized. A thorough understanding of the relationship between probiotics and the skin microbiome is important for designing novel therapeutic approaches in using topical probiotics.

## 1. Introduction

Probiotics are live microorganisms which when taken in an appropriate amount help in maintaining good health. The naturally occurring microorganisms typically constitute the first-generation probiotics, while the genetically engineered strains are the secondary probiotics. The importance of microorganisms in human health dates back to the era when Louis Pasteur first discovered the importance of fermentation and also brought attention to the fact that the consumption of fermented food may be beneficial for health and longevity. Oral and topical probiotics have been used for treating several skin conditions [1]. 

Microorganisms reside inside our body, in both the gut and on our skin. Commensal bacteria play a vital role in human health; it also helps in maintaining a healthy immune system. The skin microbiome comprises several species of microorganisms. Any imbalance in these microorganisms results in skin disorders. Acne, atopic dermatitis, psoriasis, and rosacea are some common skin conditions that arise due to an imbalance in the existing skin microbiome [2]. 

Probiotics are well known for their clinical applications in certain skin disorders and probiotic bacterial therapy may have a great potential in preventing and treating several skin conditions [3]. Research studies have established a link between the disturbed gut microbiome and inflammatory skin diseases, thereby increasing the potential of oral probiotics as a treatment option for skin disorders [4]. However, there is very little information and clinical studies that have studied the efficacy of topically applied probiotic products. The topical application of probiotic bacteria may help to enhance the skin’s natural barrier by having a direct effect at the site of application. This may be by the virtue of the resident bacteria and the probiotic bacteria that produce certain antimicrobial amino peptides which benefit the immune responses in the skin and help in eliminating pathogens. Some cosmetic formulations may help in fostering the normal skin microbiome by being selective in their activity [5].

Although topical probiotics have been used to maintain a healthy skin microbiome since the beginning of the 20th century, the last decade has seen a dramatic rise in commercially available topical probiotics [6]. With the increasing popularity of these topical products and the dearth of clinical trials or efficacy studies to establish their clinical efficiency, we aimed to write a detailed review on the use of topical probiotics in treating skin disorders. This review includes details about the normal skin microbiome, various skin disorders and the commonly used topical probiotic formulations to treat these skin conditions. We have also discussed functional clothing with probiotics embedded into the fabric as a future solution for protecting the skin microbiome.

## 2. The Skin Microbiome and Its Functions

Human skin is the largest organ of the human body and is home to several microorganisms. The microbiome is referred to a collective genome of microorganisms. Hence, the skin microbiome is a collection of genomes that serves to maintain a complex relationship on the skin. Similar to the gut, the microorganisms residing on the skin are responsible for several functions. However, distinct from the microbiome in the gut, nose or stool, the skin microbiome is still being studied by microbiologists and dermatologists [7].

The skin microbiome comprises two main groups of microbes. The resident microbiome is the core and fixed group which can replenish itself following any perturbations. The transient microbiome, on the other hand, has a microbiome that does not permanently reside on the skin but appears for a few hours or days depending on the environment. In healthy skin, both types of microbiomes are non-pathogenic. The common phyla residing on the skin include *Actinobacteria*, *Firmicutes*, *Proteobacteria* and *Bacteroides*. The three most common genera are *Corynebacteria*, *Propionibacteria* and *Staphylococci*. Each microenvironment on the skin will harbor its microorganisms. While the sebaceous follicles, which is an anaerobic, lipid-rich environment, harbors the *Propionibacterium*, the axillar area mainly consists of gram-positive bacteria of the genera *Staphylococcus*, *Micrococcus*, *Corynebacterium* as well as *Propionibacterium*. The *Staphylococcus*, *Propionibacterium*, *Micrococcus*, *Corynebacterium*, *Enhydrobacter* and *Streptococcus* species mostly grow in the drier regions of the body [7]. *Malassezia* yeast is especially present on the scalp, with *Demodex folliculorum* a mite species. The areas of skin either fall into dry, sebaceous, or moist environments. Very little information is available about the viruses that reside on the skin. Their minuscule genomic sizes make metagenomic detection a difficult task. Fungi species such as *Malassezia* include the most common *M. globosa*, *M. restricta*, and *M. sympodialis*, which are lipophilic microbes frequently associated with sebum-rich areas of the skin. These *Malassezia* spp. Are especially prevalent in sebaceous areas. The Demodex mites (such as *Demodex folliculorum* and *Demodex brevis*), which are microscopic arthropods, are also regarded as part of the normal skin flora and are the permanent ectoparasites of humans and other mammalian pilosebaceous units. Hence, Malassezia spp. And Demodex are found inhabiting the same space on the skin [8,9]. Other fungi species such as *Aspergillus*, *Cryptococcus*, *Epicoccum* and *Rhodotorula* are present near the foot skin sites [10]. Interestingly on average, every human hand has more than 25 phyla present on the surface [11].

All of these species of bacteria, fungi, bacteroid, and other microbes that dwell in the skin utilize the limited resources of lipids and sebum on the skin and adapt to live in the dry, desiccated environment [12].

Different species have developed their strategies to adapt and thrive on the skin. *Staphylococcus* are halotolerant and utilize the salt in the sweat, while some have adapted by producing ‘adherens’ to adhere to the stratum corneum while using protease enzyme to liberate nutrients from the stratum corneum to survive [13].

We acquire the skin microbiome at birth, and it remains dynamic throughout our life. During birth, the flora is less diverse and acquires the composition of the site of delivery whether vaginal birth or cesarean section. The skin colonization by the commensal microbes continues during breastfeeding and even further to finally achieve an equilibrium during adulthood. Skin is a unique ecosystem that hosts the microbiome. The microbiome has several interactions within and with the skin [7]. The skin microbiome performs the following functions:

- The residing bacteria provides the first line of defense against invading pathogens. The bac-terium *Staphylococcus* epidermidis secrete a protease that inhibits biofilm formation and colo-nization by *Staphylococcus* aureus in the anterior nostrils [13];
- The skin bacteria have a synergistic relationship with the skin’s arsenal. By upregulating the secretion of several defensive biomarkers, the skin microbiome boosts the skin’s immune function. For example, *S. epidermidis* activates the production of the Toll-like receptors TLR2 to amplify the keratinocytes response to pathogens. On the other hand, the same bacteria func-tions to inhibit TLR3 activated inflammation in wound healing activity and help to speed the process of wound healing [13];
- Human skin also shows the abundant presence of coagulase-negative staphylococci that through various mechanisms include the epidermal barrier environment and innate and adaptive immune systems within the epidermis and dermis. Some species and strains of this bacteria also produce beneficial products that augment host immunity by exerting specifically targeted antimicrobial, anti-inflammatory, or anti-neoplastic activity while also promoting broad innate and adaptive immune responses [14];
- The skin’s stratum corneum has endogenous urocanic acid that protects against the harmful ultraviolet radiations by a behavior similar to sunscreens [15]. *S. aureus* in the skin can convert histidine to the amino acid that further converts to trans urocanic acid, thereby offering UV protection to the skin [13];
- *Corynebacterium* species can modify the lipid composition on the skin’s surface and provide the thick sebum barrier [13];
- The coagulase-negative *staphylococcus* species produce bacteriocins that have antibacterial properties that limit the survival of pathogenic bacteria on the skin surface [14];
- *S. epidermidis* also has an antineoplastic activity that may protect the skin from cancer [16].

## 3. Skin Disorders

Most of the time, the microorganisms constituting the skin microbiome are in harmony with each other and perform their functions of protecting the skin. However, environmental stresses and other factors may cause a shift of the commensal microbes to pathogenic microbes resulting in inflammation, itching, scaling and other clinical signs suggesting an imbalance between our skin and its microbiome [17]. The disruption in the skin microbiome composition is termed dysbiosis. Functional dysbiosis affects the microbe-host interactions and results in skin disorders. The host factors such as age, sex, hygiene, use of certain medications, skin pH, sweating tendency, hair growth on the skin, sebum production, use of skin cosmetics and lifestyle play a significant role in the microbiome host interactions [10]. An imbalance in the skin microbiome results in several skin disorders due to loss of homeostasis. The most common skin conditions that are presented to primary care physicians are acne, atopic dermatitis, rosacea, psoriasis, and dandruff. These conditions affect majority of the population and are the most studied as they also affect psychosocial health [18]. Therefore, these skin conditions offer more avenues for research of probiotics for topical benefits [19].

### 3.1. Acne

Almost up to 90% of adolescents and young adults are affected by acne [20]. The pathophysiology of acne includes hyper seborrhea, follicular hyperkeratinization, increased colonization by microbes and inflammation [21]. The primary bacterium associated with this condition is *Cutibacterium acnes* (formerly *Propionibacterium acnes*) [22,23]. It colonizes sebaceous follicles that provide the bacterium with an anaerobic and lipid-rich environment. Secretion of several enzymes such as hyaluronidases, lipases and proteases, causes local injury and inflammation. The sebaceous gland produces and secretes a rich lipid secretion, which forms a hydrophobic layer on the skin that protects and lubricates hair and skin. Although the sebum has antibacterial functions, *P. acnes* hydrolyzes triglycerides present in secretions and releases free fatty acids that promote bacterial adherence by facilitating the colonization of these glands.

During adolescence several hormonal changes take place. There is an increased production of androgens including insulin growth factor-1, that is responsible for increased sebum production which in turn results in increased colonization of *P. acnes*. In addition to this, *S. epidermidis*, *S. aureus*, *Streptococcus pneumoniae*, *Enterobacter* and *Klebsiella pneumoniae* are the other bacteria that contribute to the inflammatory process by changing the expression of enzymes such as lipases and delta-hemolysins. The changes in the skin microbiome under the influence of age-related hormonal changes, stress, anxiety and lifestyle changes result in a breakout of acne on the skin [24]. 

While oral antibiotics are prescribed to treat acne in addition to isotretinoin and other topical antibacterial formulations [24], oral and topical probiotics have also been a recent addition to this treatment [25,26].

### 3.2. Atopic Dermatitis

Atopic dermatitis (AD) is a common inflammatory skin disease, affecting 15% to 20% of children and 1% to 3% of adults worldwide. The prevalence of AD has increased by 2-fold to 3-fold during the past decade in western countries [14]. Atopic dermatitis is a common term used in the medical literature, but according to The Nomenclature Review Committee of The World Allergy Organization, the name which should be used is eczema [27]. Atopic dermatitis is a chronic inflammatory skin condition that is one of the most frequent skin conditions around the world. The condition is characterized by dryness, redness of the skin followed by erythematous lesions. Quite often, people with atopic dermatitis also experience eczema, scaling of the skin and itching. Sometimes it is also associated with comorbidities such as allergic rhinoconjunctivitis and asthma [28]. The signs and symptoms of atopic dermatitis depend on age. While children are particularly at a high risk of developing atopic dermatitis, it is also seen in adolescents and adults [29].

There appears to be a tri-directional Atopic dermatitis-Skin microbiome-skin barrier relationship that marks the etiology of the condition. Studies have shown large colonies of *S. aureus* residing in the dry skin of people with this condition. The dysbiosis in the skin microbiome characterized by low bacterial diversity and increased *S. aureus* population in the skin results in flaring of the condition [29]. The excess *S. aureus* in the skin triggers the production of interleukins IL-36α and IL-1α in keratinocytes, which in turn induces IL-17 production in γδT cells, innate lymphoid cell type 3, and CD4^+^ T cells, and enhances neutrophil recruitment. In addition, *S. aureus* also produces various proteases enzymes to disrupt the keratinocytes thereby compromising the physical barrier of the skin. Furthermore, in these patients, there are deformed or irregular corneocytes due to filaggrin deficiency resulting in dry itchy skin [30]. Such skin is now open for abnormal entry of microbes into the dermis. The excess *S. aureus* also plays with the Th-2 immune responses and results in a dysregulation of the innate antimicrobial immune responses [14].

The use of a conventional line of treatment has not been very successful in treating the severity of this condition. Restoring the skin diversity is an important treatment strategy for treating atopic dermatitis [29]. A large number of immune cells triggered by commensals have several roles in restoring the integrity of the epidermal barrier [31,32]. Furthermore, the commensal-specific immune responses help provide heterologous protection against dermal pathogens. As a result, the use of commensal bacterial species in a topical formulation as a probiotic has been under several clinical studies at present [30]. Increased colonization with *Staphylococcus aureus* (*S. aureus*) and reduced bacterial diversity in skin microbiome has been identified as the promoter of atopic dermatitis. *S. aureus* aggravates the condition of atopic dermatitis. Monoclonal antibodies such as Dupilumab have shown improvement in atopic dermatitis, immune abnormalities, and epidermal barrier function by blocking IL-4Rα signaling. Studies indicate the increased Shannon diversity and decreased proportion of the genus *Staphylococcus* and *S. aureus* and an increase in *S. hominis* and *S. epidermidis* after treatment of atopic dermatitis with dupilumab therapy. Studies suggest that dupilumab therapy results in pronounced changes in the microbiome in lesional and non-lesional skin and in the nose, with significant changes in bacterial community structure favoring reduction in *S. aureus* population [33].

### 3.3. Psoriasis

Psoriasis is a chronic skin disease with an autoimmune origin and has affected almost 2.5–3.5% of the world population [34,35]. Around 20% of people with psoriasis experience red, scaling painful skin lesions accompanied by psoriatic arthritis. Psoriasis typically arises when there is a dysregulation in the immune responses of individuals who have a genetic predisposition to this skin condition [36].

The pathophysiology of the condition includes abnormal keratinocyte proliferation and immune cell infiltration in the dermis and epidermis involving the innate and adaptive immune systems, with important roles for dendritic cells and T cells, among other cells [37]. Studies carried out on the psoriatic lesions of the skin have shown a dysbiosis in the skin microbiome that is responsible for the dysregulation of the skin immune responses, thereby leading to an inflammatory condition. The lesions show the abundant presence of *Streptococcus* and a decreased level of *Propionibacterium*. This also indicates a lack of microbial diversity which in addition to dysbiosis is mainly responsible for psoriasis [36].

Studies have been carried out to explore the potential of probiotics in the treatment of psoriasis along with the conventional mode of treatment [3]. Conventional treatment of psoriasis involves topical as well as systemic medications depending upon the severity of the disease. Topical therapies include use of corticosteroids, vitamin D analogues, and retinoids. Systemic therapies involve administration of as methotrexate, retinoids, and ciclosporin are effective for patients with refractory or extensive cutaneous disease. In more extensive cases of disease phototherapy may be given. Characteristically, the microbiome in psoriasis patients exhibit a reduced population of beneficial microorganisms, viz., *Parabacteroides*, *Coprobacillus*, *Lactobacillus* spp., *Bifidobacterium* spp., and *F. prausnitzii*. Thus, restoring the gut microbiome balance has potential for the effective management of psoriasis. Oral administration of *Lactobacillus pentosus* significantly reduced the erythema, scaling, and epidermal thickening associated with psoriasis in animal model. [38] Reduced expression of inflammatory cytokines viz., expression of TNF-α, IL-6, and proinflammatory cytokines in the IL-23/IL-17 cytokine axis on the administration of probiotics suggest a possible therapeutic modality to manage psoriasis [39]. The administration of *L. sporogens* has also significantly improved severe pustular psoriasis in patients, who were unresponsive to conventional therapy. Encouragingly, these patients exhibited complete remission after 4 weeks of probiotic therapy [40]. However, topical use of probiotics is not yet explored for management of psoriasis. Animal studies have confirmed the effect of probiotic *Lactobacillus sakei proBio-65* extract on the severity of skin inflammation due to psoriasis in a Mouse Model [41].

### 3.4. Rosacea

Rosacea is another chronic inflammatory skin condition that occurs due to an interplay between genetics, immune dysregulation, presence of pathogenic microorganisms and neurological dysregulation. As high as 18% of the global population suffers from this skin condition. The condition is characterized by flushing and erythema as the first signs that may appear during younger ages. Telangiectasias appear as rosacea lesions in older ages. The overall rosacea manifestations are flushing, transient or persistent erythema, telangiectasia, papules, pustules, phymata, and microedema [42].

Multiple stimuli are responsible for increased levels of cathelicidin and kallikrein 5, TLR2 matrix metalloproteinases, and mast cells within the skin. The condition further progresses due to the presence of unwanted microorganisms and exposure to harmful UV radiations [43]. Emotional stress, hot and dry weather, extreme cold, alcohol, beverages and certain medications can trigger the skin condition.

Rosacea has been found to be associated with alterations in skin microbiome. Microbial examination of the rosacea-affected skin reveals colonization of the *demodex* species excess presence of *S. epidermidis* [44]. An overgrowth of commensal skin microorganisms has been observed in the skin of rosacea patients. *Demodex folliculorum* were found to be in higher density which can increase from 0.7/cm^2^ (in control subjects) to 10.8/cm^2^ in rosacea patients. Additionally, the role of *Bacillus oleronius* and *Staphylococcus epidermidis* is also suspected in causing rosacea. Interestingly, rosacea severity increased with age and the relative abundance of *C. acnes* decreased, whereas the relative abundance of *Snodgrassella alvi* increased. *Geobacillus* and *Gordonia* has also been found to be significantly associated with severity of rosacea. [45,46] Other microorganisms such as *Helicobacter pylori*, and *Bartonella quintana* are also associated with the condition. Patients with rosacea demonstrate increased densities of Demodex mites (both *D. brevis* and *D. folliculorum*) in their skin compared with controls. *D. folliculorum*’s exoskeleton itself incites the production of inflammatory markers that activates the innate immune system [47]. The products from the microbes are responsible for activation of the TLRs and the G-protein-coupled receptor proteinase-activated receptor 2 that are expressed by keratinocytes. Activation of both receptors promotes the activation of cathelicidin an anti-microbial peptide that is also overexpressed in rosacea. Increased cathelicidin levels in rosacea patients affect both vasoactive and pro-inflammatory properties [48]. Activation of TLR2 releases pro-inflammatory cytokines, chemokines, proteases, and pro-angiogenic factors, which are mediators associated with rosacea symptoms such as erythema, telangiectasia, or inflammation or a combination of these symptoms. This affects the integrity of the skin barrier resulting in more inflammation [42]. Additionally, the endobacterium *B. oleronius*, which resides inside *D. folliculorum*, secretes heat-shock proteins and a lipoprotein. It is understood that these proteins stimulate TLR2 and therefore can cause inflammation [49]. The condition is treated by symptom-based treatment modalities. A case study where scalp rosacea has been treated with oral probiotics has been reported [50]. The use of topical probiotics in the treatment of this condition needs proper clinical trials. 

### 3.5. Dandruff and Seborrheic Dermatitis

Dandruff is a skin condition affecting almost 50% of the world population. The severe form of dandruff may affect the skin by causing seborrheic dermatitis. The fungal species in the skin microbiome play an important role in dandruff and seborrheic dermatitis*. Malassezia restricta*, *Malassezia furfur*, and *Malassezia 6eborrh* are the most abundant species of the *Malassezia* genus [45]. The excess *Malassezia* spp. Growth on the skin results in inflammatory responses and results in seborrheic dermatitis [51]. Excess growth occurs in the sebum-rich area of the skin and scalp. Studies have shown bacterial imbalance in *Cutibacterium* and *Staphylococcus* species in this condition. While the conventional treatment involves antifungal, antibacterial shampoos, studies on probiotics efficiency in this condition due to the involvement of gut microbiome imbalance are being carried out [45]. Keratolytic agents or keratinization regulators are commonly used to treat dandruff conditions. These agents are applied topically. There are no studies reported for the interaction of keratolytic and probiotics. A patent (EP2149368B1) describes use of *Lactobacillus* spp. For management of dandruff. *Lactobacillus paracasei* showed positive effects in reducing the dandruff by increasing the hydration and restoring the barrier function of the scalp [52].

In addition to these commonly observed skin conditions, some skin cancer patients have also reported having an imbalance in the skin microbiome, suggesting new avenues for treating skin cancers [45].

## 4. The Importance of Gut-Skin Axis in Skin Disorders

The gut has its microbiome similar to the skin. Several research studies link inflammatory skin diseases with the imbalanced gut microbiome. The gut microbiome influences the human immune system. The immune system protects against exogenous pathogens. If there is dysbiosis in the gut microbiome, then the altered gut microflora may result in autoimmune and inflammatory conditions not only in the gut but also in distant organs such as the skin [4]. Several studies support the concept that an imbalance in the gut microbiome may result in skin conditions such as acne [53,54], atopic dermatitis [55], psoriasis [56] and rosacea [57]. The connection between the skin and gut seems to be mediated by the host immune system. The interaction between the microorganisms and the host immune system is important to maintain skin homeostasis [45]. Therefore, balancing the skin microbiome is a logical approach in treating several skin conditions. Probiotics play a vital role in restoring the microbiome and are an important therapeutic modality in the treatment of inflammatory skin diseases [4].

## 5. Role of Topical Probiotics in the Skin Microbiome

Scientists continue to explore the gut-skin axis connection in various skin disorders. With our growing knowledge about the role of the microbiome in various skin diseases, modulating the immune system by restoring the balance in the microbiome becomes a new avenue of research. The direct approach to this kind of treatment involves the use of probiotics in oral and topical form [58]. Probiotics are body-friendly bacteria that restore the body’s natural flora.

Over the past decade, there has been a surge in the use of oral and topical probiotics for skincare cosmetic formulations and the treatment of skin diseases. While new products are introduced in the market several scientists are carrying out studies to determine their efficacy, mechanism of action, safety, and indications. Topical probiotics are considered as safe a treatment modality and devoid of any adverse effects, especially if compared to conventional treatment options for skin disorders [1,59]. However, there are a limited number of clinical trials and efficacy studies in this regard. Most topical preparations are still being used as personal beauty products and the clinical trials are ongoing [60,61].

Although the precise mechanisms by which probiotics improve skin health are not yet known, the beneficial effects on skin health have been demonstrated in several papers and patents and the literature continues to grow. Table 1 lists several patents related to the application of topical probiotics for skin care. 

## 6. Topical Probiotics for the Management of Skin Disorders

**Figure 1:**
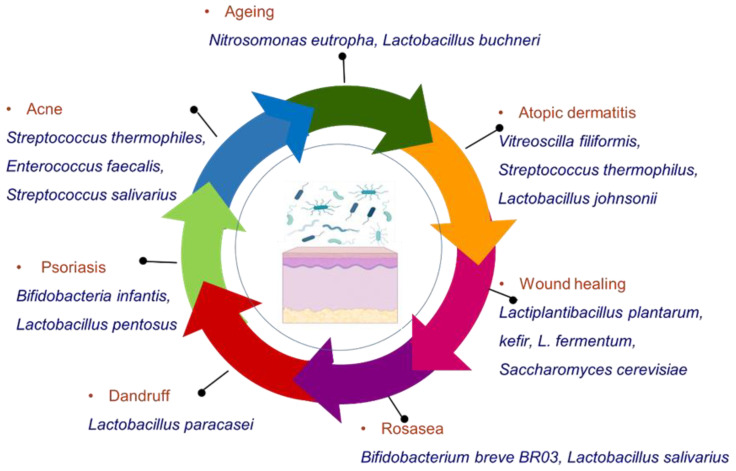
Different probiotic microorganisms useful in the management of various skin disorders.

Some studies on animals and in humans have been conducted in order to define the exact role of topical probiotics in maintaining microbial balance in skin disorders and their role in dermatology as a whole. Figure 1 represents the various probiotic microorganisms that have shown beneficial effects in the management of some common disorders.

**Figure 2:**
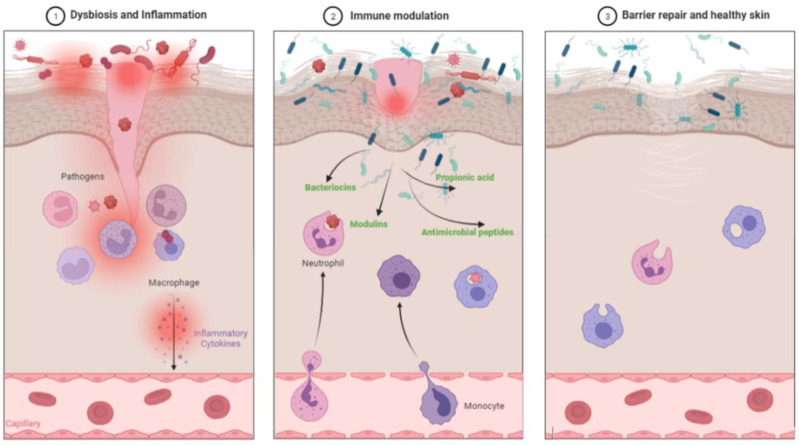
The mechanism of improving skin health by probiotics via blocking the inflammatory pathways. (1) Picture depicts dysbiosis and infection due to pathogenic bacteria (Red colored) causing inflammation. (2) Probiotic bacteria (Blue-green colored) occupy a similar ecological niche to that of pathogenic microbes and avoiding their colonization. Probiotics improve immune tolerance, reduce inflammation, and also release important biochemicals such as bacteriocins, modulins, antimicrobial peptides and propionic acid that inhibit growth of harmful microbes. (3) Recovery of the skin barrier function leading to healthy skin with healthy commensal bacteria. Following are several skin problems in which topical probiotics have shown results.

Probiotics are known to block the release of inflammatory cytokines and thus help reduce the skin inflammation [63]. Probiotics such as *L. plantarum* and *L. acidophilus* inhibit several activities of several inflammatory mediators, cytokines, and related signaling pathways. Probiotics accelerates the recovery of skin barrier function and inhibits P-induced skin inflammation [63]. We will discuss the various topical probiotics and their role in different skin disorders and also in skin aging, photoaging and wound healing. Figure 2 shows mechanism of action of probiotics in improving the skin health.

### 6.1. Acne

Individuals with acne have a unique skin microbiome. The current topical treatment for acne has several challenges since it disrupts the physical barrier of the skin, thus making it dry and irritating. Research that studies the skin-gut axis relation in acne indicated that the use of probiotics can help in improving immune reactions beyond the gut and expand them towards the skin [1]. Growing evidence suggests that topical probiotics also improved the skin barrier and produced a secondary increase in antimicrobial peptides. For example, the lactic acid bacterium *Streptococcus thermophiles* enhance ceramide production both in vitro and in vivo when applied as a cream for a week [64,65,66]. Ceramides are well-known for confining water in the skin, and certain ceramide sphingolipids such as Phyto sphingosine show antimicrobial activity against *Cutibacterium acnes*, thereby improving acne. By producing ceramides, probiotics help strengthen the skin barrier, this is beneficial to acne-affected skin as the ceramides soothe the irritated skin [67]. 

Furthermore, the topical application of phytosphingosine has been shown to reduce papules and pustules in acne patients [68]. An in vitro study evaluated the symbiotic ability of probiotic bacteria and konjac glucomannan hydrolysates to inhibit *P. acnes* growth. The authors found that different probiotic bacteria strains were able to inhibit the growth of this skin bacterium species and the presence of the GMH prebiotic significantly enhanced the inhibition [69].

In another study, a lotion containing *Enterococcus faecalis* significantly reduced pustules compared to placebo lotion. This may be attributed to the anti-*P. acnes* activity produced by *E. faecalis SL-5*. It may thus have a potential role in the treatment of acne and could be possibly used as an alternative to topical antibiotics [70].

While one study on *Streptococcus salivarius*, a prominent member of healthy humans’ oral microbiota shows inhibition of *P. acnes* [53,54,71], and some studies indicate that certain probiotic strains also showed inhibition of *E. coli*, *P. acnes*, and *P. aeruginosa* in vitro studies [72].

Hence, topical probiotics may act as protective barriers, inhibit acne-causing bacteria, reduce the pustules, and provide relief from skin irritation in acne patients.

### 6.2. Atopic Dermatitis

Atopic dermatitis primarily arises due to a reduction in the microbial diversity; the predominant microorganism in these patients is *S. aureus*, as discussed above. Several recent studies indicate that topical probiotics, in addition to emollients, may be a good alternative for treating the condition [73]. A study suggested that a 5% *Vitreoscilla filiformis* extract-containing ointment significantly reduced eczema associated with atopic dermatitis and also reduced the severity of the symptoms in a randomized, double-blind, vehicle-controlled trial [74]. Another study showed the effectiveness of the lactic acid bacterium *Streptococcus thermophilus* on the stratum corneum by improving ceramide concentrations in the skin [64]. In a randomized double-blind trial on individuals with atopic dermatitis, the researchers studied the application of a *Lactobaciilus* containing emollient as compared to a normal emollient application. The emollient containing *L. sakei* inhibited the growth of *S. aureus*, provided a physical barrier and showed improved symptoms in individuals with atopic dermatitis [75]. A trial investigating the effects of a lotion containing the heat-treated probiotic strain *Lactobacillus johnsonii NCC* on *S. aureus* colonization depicted an improvement in the clinical symptoms of individuals with atopic dermatitis. Similarly, another trial that studied the use of *Roseomonas mucosa* as a form of treatment was associated with a significant reduction in disease severity, topical steroid requirement, and *S. aureus* burden. The trial reported no adverse effects or complications [76]. Most trials conducted to date have indicated a positive effect of topical probiotics in individuals with atopic dermatitis. 

### 6.3. Psoriasis

Psoriasis, an autoimmune chronic skin condition, has been treated with topical emollients and oral immunosuppressants. Very few studies that include topical probiotics for treating psoriasis have been conducted. While studies have indicated that the alteration in the skin microbiome may be useful in controlling psoriasis symptoms, oral probiotics have shown improvement in the clinical symptoms in some studies. However, research work that studied the efficacy of topical probiotics in individuals with psoriasis is required to clinically prove the effectiveness of topical probiotics [58]. 

### 6.4. Seborrheic Dermatitis

Excess growth of yeast on the scalp with reduced diversity of microbiome results in dandruff and seborrheic dermatitis. Some studies have been carried out to evaluate the use of topical probiotics in this condition. A research study carried out on 60 patients showed a reduction in erythema, scaling and pruritis after topical application of *Vitreoscilla filiformis* [74,77]. Another study revealed increased IL-10 production by dendritic cells and increased Treg activity due to *Vitreoscilla filiformis* lysate [78]. Dandruff, seborrheic dermatitis and scalp related conditions have shown improvement after oral administration of *Lactobacillus paracasei*. There is a need for more studies on the topical efficacy of probiotics to treat this condition [79].

### 6.5. Wound Healing

Wounds due to injury to the skin result in the alteration of the skin microbiome. Studies have been carried out to find the efficacy of topical probiotics in infection prevention during wound healing [1]. Effect of *Lactiplantibacillus plantarum*, *kefir*, *L. fermentum*, and *Saccharomyces cerevisiae* has been studied in animal models with thermal wound injuries, diabetic ulcers, and uninfected wounds. Out of the models studied, mixed results were obtained. Some studies reported a positive wound healing effects by increased granulation tissue deposition, improved collagen concentration, and stimulation of angiogenesis, while some models did not improve the wound healing process [25,26].

In another study, Kefir, although originally a cultured probiotic beverage, has also shown topical antimicrobial activity against *Salmonella*, *Helicobacter*, *Shigella* and *Staphylococcus*, and *E. coli*. The probable mechanisms for this activity may be the production of exopolysaccharides that have immunostimulatory activity, activation of macrophages and lymphocytes [80,81], species-specific antagonism and through the regulation of antimicrobial peptides that help to maintain the integrity of the skin. They modulate skin microflora, improve skin integrity, and decrease inflammation [25,26]. Topical probiotics may also have the potential to inhibit the formation of biofilm over the wounds. An in-vitro study suggests commensal organisms antagonize the pathogenic strains that may produce a biofilm. [82] More clinical trials are required to prove the efficacy of topical probiotics in wound healing and for improved reproducibility of results [26].

### 6.6. Photoaging and Skin Aging

Topical probiotics have made the latest entry into the skincare world with their use in photoaging and skin aging. While the clinical trials for the same are underway, there has been a study that depicts the use of probiotics in slowing the process of photoaging, reducing oxidative stress and improving the barrier function of the skin [83]. Another research has studied plant extracts fermented with *Lactobacillus buchneri* for photoaging effect in the in-vitro models [70]. One study shows the use of topical *Nitrosomonas eutropha* to treat facial wrinkles. The researchers have found a significant improvement in wrinkle-depth severity, hyperpigmentation of the forehead and glabella in the group receiving high topical concentration of the probiotic formula [84].

### 6.7. Rosacea 

Rosacea arises due to overexpression of TLR2 receptors leading to inflammatory reactions and altered skin microbiome [25,26]. Scalp rosacea has been treated using an oral probiotic in addition to doxycycline as antibiotic, however, topical probiotics have not yet been explored for rosacea treatment [50].

## 7. Marketed Topical Probiotics Skin Care Products

While the use of topical biotherapy dates back to 1912 when the topical application of *Lactobacillus bulgaricus* improved the conditions such as acne and seborrhea, the skincare industry has recently seen a surge in the topical formulations containing microorganisms [6,19]. Various different skincare products containing probiotics marketed globally are listed in Table 2. 

## 8. Recent Novel Approach in Topical Probiotics: Probiotic Coated Apparels

The hospital clothes, aprons, gowns, and other apparel worn by the hospital staff and patients are contaminated with microorganisms which increases the chances of hospital-acquired infections. Although the incorporation of antimicrobials has been used in wash liquids to kill the pathogens in the clothes, the use of probiotics in hospital clothing is a novel approach in using topical probiotics for skin infections and conditions. In one such study, the researchers have incorporated probiotics in the form of paste in the fabric of the hospital apparel that stayed even after five washes. Including probiotics in hospital linens and uniforms may protect against infections [85]. Another study successfully demonstrated probiotics printing using a paste containing probiotic spores on a polyester fabric. The probiotic printed fabric had higher contact angles and lower wettability [86].

Such clothing with probiotics embedded in the fabric may be the future of functional clothing to combat nosocomial pathogens and to treat infections. While it positively interacts with the skin microbiome, it also helps in keeping pathogens at bay and helps to handle skin odor, skin conditions and infections [87].

## 9. Regulatory Aspects of Probiotics in Topical Skin Care and the Challenges

Since there are several potential applications for probiotics in management of skin related disorders, a proper regulation of the labelling and marketing standards is essential. Almost all of the probiotic-containing topical formulations have yet not gone beyond the personal care product category. Additionally, these topical care products are non-sterile and may contain antimicrobial preservatives that may affect the probiotic strain viability and further alter the microbiota of the recipient in an already diseased state [84]. Therefore, regulations of probiotics primarily need to be concerned with safety. At present, there are no specific guidelines for commercializing probiotics, and products are regulated according to their final use either as a drug, medical device, food, dietary supplement, or cosmetic. Probiotic-based products with health claims could be notified as pharmaceuticals or medicines [88]. If the product contains non-viable microorganisms, it will be considered as a medical device. Probiotic skin care products are a very recent innovation and there is a lack of scientifically validated clinical data about the efficacy and safety of topically applied probiotics. Additionally, the sharp demarcation between foods, drugs, and cosmetics, makes the development and notification of probiotic cosmetic products a challenge.

Topically applied products are not produced in sterile conditions and hence a sterility test is not required. These products often contain preservatives to control the growth of microbes. These preservatives can potentially affect the probiotic strain viability and also inadvertently alter the microbiota of the recipient [19]. Probiotic-containing topical formulations have yet not gone beyond the personal care product category; since they have high load of colony forming units, it is difficult for such preparations to pass the USFDA regulatory requirements for microbial load. Preservative effectiveness test is a major hurdle in testing these products. Topical probiotic formulations for treating acne were tested for microbial enumeration testing as per United States Pharmacopoeia (USP). It was found that topical products do not contain “objectionable” quantity of live microorganism and hence is not required to be below 1000 CFU as per USP [60].

Performing the bacterial challenge test is a big challenge for preparations containing live bacteria. In 1 particular study, blank formulations were put through challenge tests for 14 and 28 days. The blank formulation showed a decreased load of inoculated bacteria and no increase in yeast or mould after 14th and 28th day of challenge test. The results proved that in that particular formulation, low water activity is a reason for self-preservation of the formulation and no external preservative is needed [89].

## 10. Concluding Remarks

In recent years, significant advances have been made in understanding the composition of the skin-microbiome and how the dysbiosis affects the skin health. Topical probiotics in the form of various dermatological formulations are an important part of the treatment of skin conditions. While the skin microbiome, its functions and protective nature maintain the skin homeostasis, an imbalance results in inflammatory skin conditions that are difficult to get completely cured by conventional treatments. Several clinical trials are being carried out in order to study the efficacy as well as the adverse effects of topical probiotic formulations for the treatment of conditions such as atopic dermatitis, acne, psoriasis, wound healing and many other skin problems. We hope that this review forms a contribution to promoting enhanced research activities in the field of topical probiotics as a novel therapeutic approach for the treatment of skin disorders.

## Figures and Tables

**Table 1:** Several patents related to the use of topical probiotics for the treatment of various skin disorders.

Sr. No.	Title of Invention	Probiotic Strain Used	Use of the Product	Date of Publication	Patent Number
1	Cosmetic composition having probiotic bacteria	*Bifidobacteria longum; Bifidobacteria bifidum; Bifidobacteria breve; Bifidobacteria pseudolongum*, or mixtures thereof	Cleaning and antiageing treatment	20 April 2017	WO2017063066A1
	Skin probiotic	Probiotic commensal skin bacteria, and probiotic commensal skin bacteria fermentation extract. The probiotic commensal skin bacteria from one of a Propionibacterium species, a Paenibacillus species, or a Staphylococcus species.	For producing or maintaining skin microbiome balance	16 July 2015	WO2015106175A1
3	Composition, topical probiotic, postbiotic, pharmaceutical and topical compositions, methods for treating skin or mucosal infections, for treating a dermatological disorder associated with c. acnes and for diagnosing a skin disease or disorder, and formulation for topical application	*S. capitis* N030_E12, *S. epidermidis* AMT5_C5, *S. epidermidis* N009_G7, *S. epidermidis* N018_F3	For the topical treatment of dysbiosis disorders of the skin, scalp or mucous membranes; and acne.	13 September 2019 19 March 2020	BR112021004538A2 WO 2020/056359
4	Method for inhibiting the growth, migration, proliferation and/or metastasis of a precancerous, cancerous or neoplastic cell or inhibiting a pathogen, topical probiotic composition, bandage or dressing, method of treating skin damage due to UV radiation and pharmaceutical composition	*S. epidermidis* MO34 or MO38 or MO34 and MO38	Useful for treating infections, cancer and neoplastic diseases and neoplastic disorders.	27 March 2018 4 October 2018	BR112019017962A2 WO 2018/183288
5	Skin microbiome colonizer formulations and methods for use	*Bacillus licheniformis*, *Bifidobacterium breve*, *Bifidobacterium infantis*, *Lactobacillus fermentum*, *Lactobacillus plantarum*, *Lactobacillus rhamnosus*, *Lactobacillus sakei*, *Lactobacillus paracasei*, *Staphylococcus epidermidis*, and *Staphylococcus xylosus*.	For treating dysbiosis of the skin	30 June 2020	US10695386B2
6	Probiotic treatment of skin diseases, disorders, and infections: formulations, methods and systems	*Lactobacillus jensenii*, *Lactobacillus vaginalis*	For reducing dandruff of the scalp	26 March 2019	US10238597B2
7	Method of using topical probiotics for the inhibition of surface contamination by a pathogenic microorganism and composition therefor	*Lactobacillus acidophilus* NCFM^®^; *Lactobacillus acidophilus* La-14; *Lactobacillus paracasei* Lpc-37	Prevent contamination by pathogenic microorganisms	8 May 2008	US20080107699A1
8	Cosmetic composition comprising cocoa bean hydrolysate and at least one prebiotic and probiotic	Lysate of the genus Bacillus	Treatment and/or alleviation of seborrheic disorders, in particular sebum-dependent inflammation and/or bacterial proliferation.	28 August 2020	CN111601583A
9	Use of probiotic microorganisms to limit skin irritations	*Lactobacillus paracasei* species	To prevent skin irritation due to external aggressions	2 April 2020	ES2751994T3
10	Cosmetic and dermatological use of probiotic microorganisms Lactobacillus paracasei for the treatment of fatty disorders of the scalp	*Lactobacillus paracasei*, or a lysate thereof.	Treatment of fatty disorders of the scalp	7 August 2018	ES2677905T3
11	Cosmetic composition comprising a eborrheic spinosa extract, a kappaphycus alvarezii extract, at least one prebiotic and a probiotic	Lysate of Lactobacillus bacteria	To maintain the balance of the cutaneous microbiota, and reinforce the cutaneous barrier	28 October 2020	EP3727594A1
12	Cosmetic and/or dermatological composition for preventing and/or treating sensitive or dry skin	*Lactobacillus johnsonii* (CNCM I-1225), *Lactobacillus paracasei* (CNCM I-2116), *Bifidobacterium adrecentis* (CNCM I-2168), *Bifidobacterium longum* (CNCM 1-2170)), *Bifidobacterium lactis* (CNCM I-3446), *Bifidobacterium longum* (BB536)	Prevention and/or treatment of sensitive and/or dry skin	29 January 2009	JP2009503042A
13	Antibacterial lysate of probiotic bacteria	*Lactobacillus rhamnosus*	For skin tissue repair, wound repair	12 March 2015	WO2015/181534 A1
14	Probiotic stick formulation for skin maintenance and methods of use	*Streptococcus thermophilus*, *Streptococcus salivarus* subspecies *thermophilus* type 1131, *Bifidobacterium longum*, *Lactobacillus paracasei* and *Streptococcus salivarius*.	Hydration of skin, reduction in lines or wrinkles, treating acne and skin inflammation	16 May 2017	US9649346B2
15	Probiotic bacteria for the topical treatment of skin disorders	Cell lysates from *Bifidobacterium*, *Brevibacterium*, *Propionibacterium*, *Lactococcus*, *Streptococcus*, *Lactobacillus (e.g*., *L. acidophilus)*, *Enterococcus*, *Pediococcus*, *Leuconostoc*, *and/or Oenococcus*	Improvement of tight junction integrity between normal human epidermal keratinocytes.	26 September 2018 08 November 2012	EP 2 704 704 B1 WO 2012/150269
16	Topical compositions containing probiotic bacillus bacteria, spores, and extracellular products and uses thereof	*Bacillus coagulans* *Pseudomonas lindbergii*	Inhibit the growth of bacterium, yeast, fungi.	20 April 2008	US6723326B1
17	Use of cosmetic agent of probiotic microorganism in particular of Lactobacillus and Bifidobacterium species genus, for treating scalp disorders e.g., dandruff condition, pruritis or eborrheic dermatitis	*Lactobacillus or bifodobacterium*	Restoration of normal scalp, reduces irritation, itching and dandruff	21 May 2010	FR2938429B1
18	Use of Lactobacillus plantarum gmnl-6 composition for skin care	*Lactobacillus plantarum*	Improving skin condition by collagen secretion and ceramide synthesis	18 May 2021	US11007137B2
19	Topical use of probiotic bacillus spores to prevent or control microbial infections	*Bacillus* species, spores or an extracellular product of a *B. coagulans* strain	inhibiting growth of yeast, fungus, bacteria or Herpes simplex virus	9 November 2009	EPO975227A1
20	Use of probiotic bacteria for the preparation of topical compositions for skin protection	*L plantarum P 17630*	Preventing and treating microbial diseases of the skin	9 February 2006	WO 2006/013441A2

There are a few examples wherein targeted approaches have been used for the management of acne and rosacea. E.g., Use of antibacterial peptides produced by *Staphylococcus* to reduce the burden of *S. aureus* and increase diversity in atopic dermatitis [46,62].

**Table 2:** Commercially available skincare products throughout the world.

Sr. No	Brand/Manufacturer	Type of Skin-Care Product	Probiotic Used	Benefits Claimed
1	Okana	Eye care serum	*Bacillus* bacterial ferment extract	Helps skin retain its firmness and elasticity and keeps it feeling smooth and plump.
2	Amperna	Probiotic+ Ds Soothing Serum	Unique probiotic complex	Soothe irritated skin and calm redness. Tested on eczema, dermatitis, perioral dermatitis, rosacea and acne-prone skin.
3	Elissah Bio P2 Laviol Skin Care	All in One Essence, triple action, concentrated topical facial serum	16 types and 35 strains of bacteria including 14 *Bifidobacterium* and *Lactobacilli*.	Strengthen the skin’s barrier against environmental threats and reduce the factors that trigger skin sensitivities, redness and irritation.
4	Probiotic Skin Cream Melvory	Face Cream	*Lactobacilli* probiotic (*Lactobacillus* ferment filtrate)	Cleans away the bad bacteria on the skin. For acne-prone or teenage skin
5	Andalou Brightening Probiotic + C Renewal Cream	Cream	*Bacillus coagulans*	Skin-friendly vegan probiotic microflora enzymatically supports dermal vitality, targeting over-exposed surface cells for a lighter, tighter, brighter looking appearance and a luminous complexion.
6	Biossance Squalane + Probiotic Gel	Face moisturizer	*Lactococcus* ferment lysate	Help restore the skin’s balance and renew the skin barrier
7	Neogen Dermalogy Probiotics Double Action	Facial Serum	The patented complex of *Bifida ferment lysate*, *Lactobacillus*, and *Streptococcus thermophilus* ferment	Protect the skin barrier
8	Elemis Dynamic Resurfacing Facial Pads	Facial Pads	*Lactococcus* ferment lysate	Stimulate skin-cell renewal and reinforce the skin barrier
9	Missha Time Revolution—The First Treatment Essence Rx	Toner	*Bifida* ferment lysate	Promotes better product absorption, as well as moisture retention and elasticity of the skin.
10	Éminence Organic Skin Care Clear Skin Probiotic Masque	Face mask for oily skin	Lactic acid bacteria derived from yogurt	Exfoliates and moisturizes the skin. Improve the skin’s moisture absorption and ward off the signs of aging. Reduce the intensity of wrinkles and tightens pores.
11	Manyo Factory Bifida Complex Ampoule	Facial serum	*Bifida* ferment lysate, *Bifida* ferment filtrate, *Lactobacillus* ferment lysate, and *Lactococcus* ferment lysate	Encourages self repair of skin, hydrates, replenishes moisture and prevents aging
12	La Roche-Posay Lipikar Balm AP+ Intense Repair Moisturizing Cream	Cream for dry skin	APF, a patented version of the waterborne bacteria *Vitreoscilla filiformis*	Improve severely dry skin and keep it hydrated.
13	Mother Dirt AO+ Mist	Toner for oily, acne-prone skin	The soil-derived *Nitrosomonas eutropha* bacteria	Mist protects and maintains healthy skin by restoring and balancing your microbiome
14	LaFlore Probiotic Serum Concentrate	Facial serum for sensitive, irritated, and rosacea-prone skin	*Lactococcus* ferment lysate and live kefir probiotics (*Hansenula/Kloeckera/Lactobacillus/Lactococcus/Leuconostoc/Pediococcus/Saccharomyces*)	Helps calm and smooth fine lines and wrinkles and boosts the skin’s natural defense system.
15	Marie Veronique Pre+Probiotic Daily Mist	Toner for all types of skin	A proprietary microbiotic complex that contains a combination of 34 probiotic live strains including a high concentration of *Bifidobacterium*	Balances the skin’s microbiota, thereby reducing skin irritations that lead to inflammation
16	Columbia Skincare Probiotic Concentrate	Serum for sensitive, irritated, and rosacea-prone skin	*Lactococcus* ferment lysate	Enhance skin’s immune function
17	Elizabeth Arden Superstart Probiotic Boost Skin Renewal Biocellulose Mask	Face mask for dull and damaged skin	*Lactococcus* ferment lysate; inactivated strains of *Lactobacillus casei* and *Lactobacillus acidophilus*	Optimizes skin’s microflora and natural defence. Moisturizes and smoothes skin.
18	Glowbiotics HydraGlow Cream Oil	Hydrating oil for dull, sun-damaged skin	*Lactococcus* ferment lysate	Stimulates the skin’s renewal process
19	FCL Pre+ Probiotic Body lotion	Body lotion for skin nutrition, improving skin protective barrier and provides moisture	Probiotic *bifido* culture	Detoxifies skin, provides natural skin repair, promotes microbiota balance and hydration.
20	Herbostra Probiotic hand cream	Non-greasy hand cream to overcome dryness	Probiotic enzymes	Moisturizes and nourishes hands, nails, cuticles. Rich and smooth texture with antimicrobial properties keep hands moisturized.
21	Dot and Key 72 h hydrating gel and Probiotics	Hydrating, oil-free gel for dry parched skin	*Saccharomyces* black tea ferment, *lactobacillus*	Provides long hours of moisturization, restores microbiome balance
22	Tula Instant Depuff eye renewal serum	Balm available as a stick	Yogurt Powder, Algae Extract, *Bacillus* Ferment, *Lactococcus* Ferment Lysate	Ideal for aging skin, crow feet near eyes, under-eye dark circles, and eye puffiness.
23	Ren Perfect Canvas clean primer	Hydrating drops	*Lactococcus* Ferment Lysate	Minimizes skin pores and fine lines. Creates a base for a perfect make up
24	Clinique redness solutions daily relief cream.	Oil-free moisturizing cream	*Lactobacillus*	Restores microbiome and skin barrier, reduces redness and irritation of the skin by calming it.
25	Pretty pop probiotic power whipped cream	Whipped cream with encapsulated probiotics	*Lactobacillus* Ferment, *Lactococcus* Ferment Lysate, *Bifida* Ferment Lysate, *Lactobacillus*, *Streptococcus thermophilus* Ferment,	Treats dryness, wrinkles, fine lines, loss of elasticity and firmness.
26	Korres Greek Yoghurt Skin nourishing probiotic gel-cream	Gel-cream for dryness and redness of the skin	*Lactobacillus*, Greek yogurt, yoghurt powder	Nourishes, hydrates and gives a bouncy feeling skin.
27	Edible beauty probiotic radiance tonic	Serum to soothe skin from redness and irritation	*Lactobacillus* Ferment Filtrate	Reduces redness, aging effects and irritation of the skin
28	Glamglow berry glow probiotic recovery mask	Recovery mask packed with super berries and probiotics	*Lactobacillus* Ferment	Balances and supports skin microbiome, replenishes hydration, and protects skin moisture barrier.
29	Korres Probiotic Superdose facemask	Facemask containing Greek yoghurt and pre and probiotics	*Lactobacillus*, Greek yogurt	Nourishes and replenishes the skin, restores microbiome
30	Beekman 1802 Bloom Cream daily moisturizer	Daily moisturizing cream for glowing radiance	*Bifida* Ferment Lysate	Microbiome friendly, hydrates skin, gives healthy youthful glowing skin
31	Kinship Self Reflect Rose Probiotic Moisturizing Sunscreen Zinc Oxide SPF 32	Probiotic moisturizing sunscreen cream	*Lactobacillus* Ferment, Plant-based probiotics	Works as a moisturizer, primer and Sunscreen with broad-spectrum UV protection
32	Clinique Redness Solutions Makeup Broad Spectrum SPF 15 With Probiotic Technology Foundation	Oil-free liquid foundation with SPF	*Lactobacillus* Ferment	Covers flushing. Blushing, redness and irritation of the skin. Protects against UV radiation
33	Beekman 1802 Milk Drops Probiotic Ceramide Serum	lightweight probiotic milk serum	*Bifida* Ferment Lysate	Moisturizes and restores the skin, microbiome friendly formula that boosts the dull skin, cleans pores, helps control oil
34	Beekman 1802 Mini Milk Wash Exfoliating Jelly Cleanser	a light jelly formula that transforms into a deep-cleansing milk	*Bifida* Ferment Lysate	Deeply cleanses and exfoliates for clearer, brighter skin, helps control oily skin, clears pores, and fights blemishes
35	Beekman 1802 Milk Bar Probiotic Facial Cleansing Bar	Solid bar that transforms into foaming cleanser with little water	*Bifida* Ferment Lysate	Deeply cleanses skin with ultra-foaming lather
36	Ulta probiotic cream mask	Cream mask	Yogurt Extract, *Lactobacillus*	Nourishes, clarifies and soothes skin. It also conditions and tones the skin
37	WLDKAT Prebiotic + Probiotic Pleasure Serum	Water-based intimate serum for vaginal use	*Lactobacillus* Ferment,	Fights vaginosis, maintains natural moisture, pH, enhances pleasure and has antibacterial properties
38	Skyn Iceland Nordic Renewal Pre + Probiotic Cream Starter Set	Pre + Probiotic Cream	three potent fermented extracts of probiotics	Skin renewal, enhances skin microbiome, deeply nourishes and moisturizes
39	Boscia Prebiotic + Probiotic Freshening All-Over Body Deodorant	Body deodorant	*Lactococcus* Ferment Lysate	Neutralizes odor, restores skin balance, combats wetness and soothes for clean, long-lasting protection
40	Pacifica Sun + Skincare Mineral Face Shade Coconut Probiotic SPF 30	Moisturizing face lotion that applies evenly and leaves skin hydrated	*Lactococcus* Ferment Lysate	Provides water-resistant protection with a sheer application.

## Data Availability

Not applicable.
